# Polyvinyl Alcohol/Lignin Composite Films Crosslinked with Citric Acid: Physicochemical Properties and Anti-Adhesive Activity Against *Staphylococcus aureus*

**DOI:** 10.3390/polym18182244

**Published:** 2026-09-15

**Authors:** María V. Martinez, Julieta Chiappero, Diego F. Acevedo, Cesar A. Barbero, Edith I. Yslas

**Affiliations:** 1 Departamento de Tecnología Química, Facultad de Ingeniería, Research Institute for Energy Technologies and Advanced Materials (IITEMA), National University of Río Cuarto (UNRC)-National Council of Scientific and Technical Research (CONICET), Universidad Nacional de Río Cuarto, Río Cuarto X5800, Argentina; mvmartinez@ing.unrc.edu.ar (M.V.M.); dacevedo@ing.unrc.edu.ar (D.F.A.); 2 Departamento de Química, Facultad de Cs. Exactas Físico-Química y Naturales, Research Institute for Energy Technologies and Advanced Materials (IITEMA), National University of Río Cuarto (UNRC)-National Council of Scientific and Technical Research (CONICET), Universidad Nacional de Río Cuarto, Río Cuarto X5800, Argentina; cbarbero@exa.unrc.edu.ar; 3 Departamento de Biología Molecular, Facultad de Cs. Exactas Físico-Química y Naturales, National University of Río Cuarto (UNRC)-National Council of Scientific and Technical Research (CONICET), Universidad Nacional de Río Cuarto, Río Cuarto X5800, Argentina; jchiapppero@exa.unrc.edu.ar; 4 Departamento de Biología Molecular, Facultad de Cs. Exactas Físico-Química y Naturales, Research Institute for Energy Technologies and Advanced Materials (IITEMA), National University of Río Cuarto (UNRC)-National Council of Scientific and Technical Research (CONICET), Universidad Nacional de Río Cuarto, Río Cuarto X5800, Argentina

**Keywords:** biopolymers, lignin, anti-adhesion, hydrophilic materials

## Abstract

Antibiotic-resistant infections, particularly in hospital settings, are increasingly associated with biofilm formation on biomedical devices. An effective strategy to address this problem is the development of biopolymeric films with antibacterial and anti-adhesive properties. In this work, polyvinyl alcohol (PVA), a synthetic biodegradable polymer, was composited with lignin (LG), a biomass-derived polymer, and crosslinked with citric acid (CA) to obtain PVA-LG/CA gels/films. Materials were prepared with fixed PVA content (10% *w*/*v*) and varying LG (6.6 and 10% *w*/*v*) and CA (1–4% *w*/*v*) contents. FTIR and DSC analyses confirmed the role of CA as a crosslinker through esterification with the hydroxyl groups of PVA and LG. The lignin-free film (PVA/CA) showed the lowest water swelling (%Sw = 54.7), while PVA-LG/CA films exhibited significantly higher swelling, reaching 409.5%. All films were hydrophilic (contact angle < 90°). Increasing CA reduced porosity, whereas higher LG decreased hydrophilicity. Biological assays showed that films with higher LG and CA contents reduced bacterial adhesion. Overall, the formulation with 6.6% LG and 3.3% CA displayed the best performance, combining maximum swelling and minimal *Staphylococcus aureus* adhesion. These results highlight the potential of LG and CA as tunable components for modulating film morphology, hydrophilic balance, and bacterial interactions in biomedical material design.

## 1. Introduction

Due to the substantial threat that bacterial infections pose to human health and the widespread occurrence of antibiotic resistance [1,2,3], developing novel antibacterial and anti-adhesive materials has become a major focus of biomedical research [4].

Several studies have demonstrated that certain bacteria, including *Staphylococcus aureus*, can survive on various surfaces for weeks, posing a significant risk of infection [5,6,7]. Also, microbial adhesion to surfaces is a common cause of persistent inflammatory processes and the eventual failure of biomedical devices such as vascular and urinary catheters, vascular implants, heart valves, and prostheses, underscoring the clinical relevance of developing antibacterial and anti-adhesive materials that could eventually be applied as protective coatings on such surfaces.

To address this issue, modified surfaces have been developed to reduce bacterial attachment, which has spurred significant interest in the study of polymeric films with inherent anti-adhesive properties. Among the several materials developed, polyvinyl alcohol (PVA) and lignin have been extensively studied. Also, studies have reported that PVA modified with lignin produces novel materials with enhanced mechanical and thermal properties, as well as improved ultraviolet-blocking, antioxidant, and antimicrobial performance [8].

PVA is a synthetic, semi-crystalline, hydrophilic and biodegradable polymer [9]. It is widely used in medical applications and food packaging [10]. PVA is an appealing candidate for these applications owing to its nontoxicity, outstanding biocompatibility, and solvent resistance [11]. Natural polymers have gained increasing attention mainly because of their abundance and low cost. Lignin is a natural polymer derived from plants that possesses many attractive properties, such as renewability, biodegradability, and natural antioxidant and UV protection activity. Lignin is regarded as a promising material owing to its biocompatibility, cost-effectiveness, and abundance in nature [12]. In the medical area, lignin derivatives have potential applications due to their versatility as wound dressings, pharmaceuticals, and drug delivery systems. The utilization of lignin is of significant interest since it is the most prevalent polyphenol, with diverse applications, particularly in the development of functional materials such as hydrogels [13,14]. These polymeric films can be engineered to possess intrinsic characteristics that influence microbial colonization, thereby enhancing the longevity and safety of biomedical devices. For instance, incorporating antimicrobial agents directly into the polymer matrix or altering the surface chemistry to create a hostile environment for bacteria are promising strategies. Additionally, surface modifications that make the material less prone to bacterial adhesion, such as hydrophobic or superhydrophobic coatings, have shown potential in minimizing the risk of biofilm formation and subsequent infection [15,16].

Ongoing research in this field aims to identify and optimize materials that can effectively prevent microbial attachment and growth, thereby improving the clinical outcomes of patients relying on biomedical devices.

The aim of this study is to not only design a practical biopolymeric film based on PVA-LG/CA that exhibits superior anti-adhesion characteristics, but also to study the influence of the ratio of the components of the biopolymers on these properties. The results demonstrate that varying the concentrations of LG and CA yields materials with interesting chemical and morphologic characteristics. This composition–property screening was conceived as a necessary first step before depositing the optimized formulation on a real device.

## 2. Materials and Methods

### 2.1. Materials

Polyvinyl alcohol (PVA, MW: 89,000–98,000 Da, Hydrolysis: 99%) and lignin alkali (LG, MW: 10,000 Da, pH = 10.5, purity > 96%) were purchased from Sigma-Aldrich, St. Louis, MO, USA. Anhydrous citric acid (CA, purity > 99.5%) and sodium hydroxide (NaOH, purity > 97%) were purchased locally from Cicarelli, Santa Fe, Argentina. All chemicals were of analytical grade and used as received.

### 2.2. Biomaterials Synthesis

The concentrations of PVA, LG, and CA, as summarized in Table 1, were selected based on preliminary experiments aimed at obtaining films with adequate stability in aqueous media while avoiding excessive crosslinking and rigidity. For the PVA-CA formulation (B1), CA was used at 10 wt% relative to the PVA content (1% *w*/*v*), which was the minimum concentration that produced a water-stable film under the experimental conditions. Lower CA concentrations resulted in films that progressively dissolved in water within 2–3 days, whereas higher CA contents produced more highly crosslinked and rigid materials [17]. For the PVA-LG formulations, B3 was initially prepared using the same criterion, with CA corresponding to 10 wt% of the total PVA and LG content (1.7% *w*/*v*). Since increasing the LG concentration without increasing the CA content reduced the stability of the films in aqueous media, the PVA and LG concentrations were subsequently kept constant while the CA concentration was varied (B2–B4) to obtain materials with different degrees of crosslinking. Finally, B5 was formulated with a higher LG concentration, which required increasing the CA content to 20 wt% relative to the total PVA and LG content (4% *w*/*v*) to obtain a stable film.

**Scheme 1:**
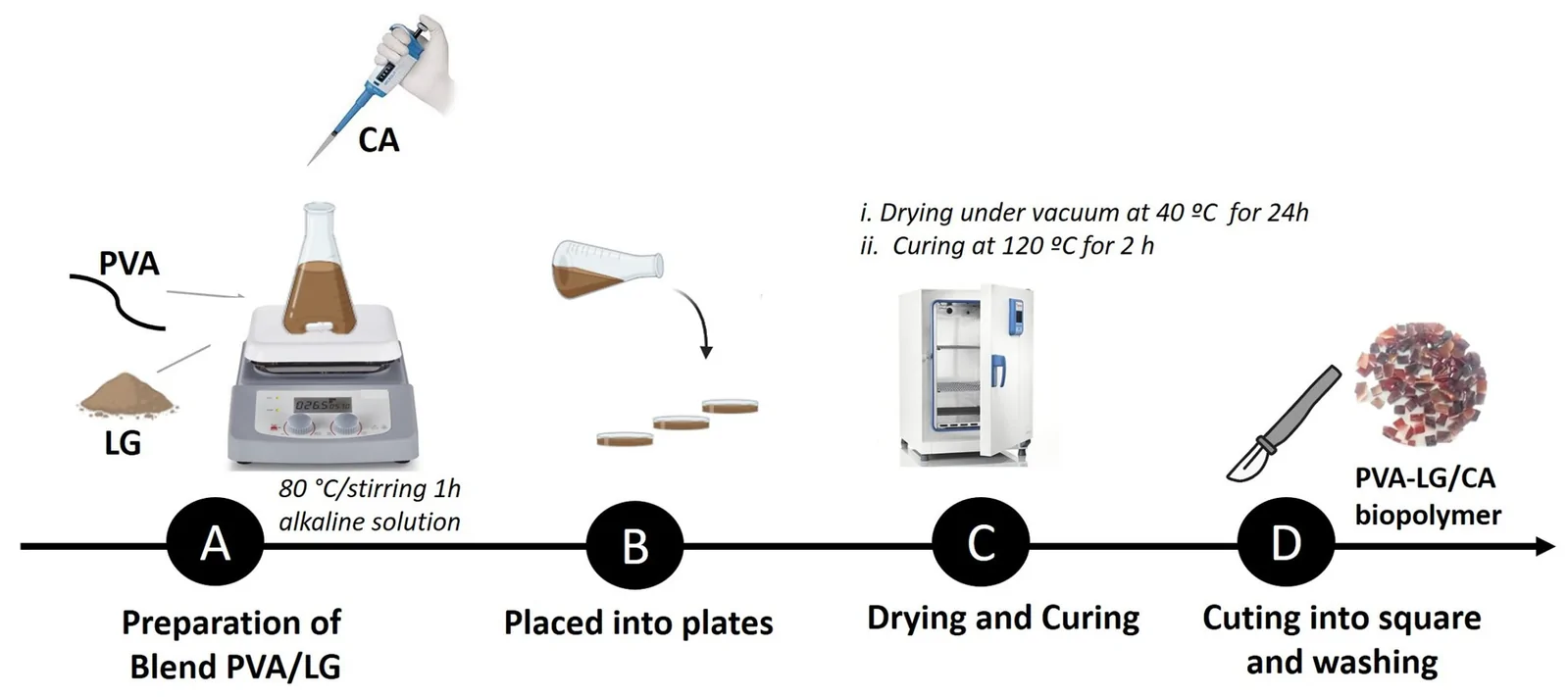
Experimental steps for the synthesis of PVA-LG/CA films.

The synthesis involved four steps: (A) First, a measured amount of pure PVA or PVA-LG was added to an alkaline solution (2% *w*/*v* NaOH) and heated up to 80 °C with continuous stirring until fully dissolved. Citric acid (CA) was then added, allowing the reaction to proceed for 1 h at the same temperature. (B) The resulting blends were cast onto glass plates. (C) The casted blends were dried at 40 °C under reduced pressure for 24 h, followed by curing at 120 °C for 2 h. (D). Finally, the films were cut into squares, washed twice with distilled water to remove any unreacted material, and subsequently dried in an oven at 40 °C for 24 h. Scheme 1 shows a summary of these synthesis steps.

To determine the residual amount of LG in each film after the washing steps, the following assay was conducted in triplicate: the films, previously cut into squares and weighed using an analytical balance, were immersed in 15 mL of distilled water (first washing) at 25 °C for 24 h. Subsequently, an aliquot of 100 μL of the washed solution was extracted, and the concentration of LG was quantified using the Folin–Ciocalteu (FC) method [18,19]. A second washing step was performed by repeating this procedure.

The FC method is a widely utilized colorimetric assay for quantifying total phenolic compounds by generating a blue-colored complex, which can be measured via spectrophotometry. The procedure for quantifying LG was adapted from that described by described by Singleton et al. [19]. Briefly, the total volume of reaction mixture was reduced to 10 mL, comprising 100 μL of the sample or standard, 500 μL of FC reagent, 1.5 mL of sodium carbonate (20% *w*/*v*), and 8 mL of distilled water. Following a 1 h incubation at 25 °C, the blue color developed completely, and the absorbance was measured at 760 nm using a Hewlett-Packard 8453 spectrophotometer (Hewlett-Packard Company, Palo Alto, CA, USA). To build the calibration curve (see Figure S1), lignin at a concentration of 2 g·L^−1^ was utilized to prepare standard solutions. The absorptivity coefficient was determined to be 8.59 L·g^−1^·cm^−1^.

#### 2.2.1. Chemical Characterization

The films were characterized using Fourier-transform infrared (FTIR-ATR) spectroscopy (PerkinElmer, Inc., Waltham, MA, USA) to identify functional groups and confirm the esterification reaction. A Perkin Elmer Spectrum Two spectrometer was used. The spectra were acquired in absorbance mode with 20 scans per spectrum at a spectral resolution of 4 cm^−1^ in the wavenumber range from 4000 to 500 cm^−1^. As a reference, the background spectrum of air was collected before the acquisition of each sample spectrum. Duplicate spectra were recorded per sample.

#### 2.2.2. Thermal Analysis via Differential Scanning Calorimetry (DCS)

The DSC analysis was conducted using a Netzsch DSC-204-F1-Phoenix (NETZSCH-Gerätebau GmbH, Selb, Germany) under N_2_ flow. First, the sample (ca. 10 mg) was quickly cooled inside the DSC chamber at −25 °C. The system was kept at this temperature until the thermal equilibrium state was reached (five minutes). Then, the sample holder assembly was heated from −25 to 450 °C at a rate of 5 °C·min^−1^.

#### 2.2.3. Morphologic Analysis by Scanning Electron Microscopy (SEM)

The biomaterials were pre-treated prior to SEM analysis using the following steps: (1) Freeze-drying: the samples were freeze-dried using a RIFICOR L-T4 freeze dryer at −60 °C and a vacuum pressure of 10^−6^ torr for 48 h. (2) Sputter coating: The samples were sputter-coated with a thin layer of gold for 90 s at a current of 40 mA and a pressure of 0.4 mbar under an argon atmosphere. Scanning electron micrographs were subsequently obtained at low vacuum and low field conditions using a Carl Zeiss EVOMA10 microscope (Carl Zeiss Microscopy GmbH, Jena, Germany).

#### 2.2.4. Swelling Capacity

To determine the capacity to incorporate water, the swelling percentage in equilibrium (%Sw_eq_) was measured as follows: dry samples were first weighed (ws) and then placed in the water solution at room temperature. After 24 h, sufficient time to achieve equilibrium, the biopolymers were removed from the solution, superficially dried with tissue paper, and re-weighed (weq) using an analytical balance. The %Sw_eq_ was calculated according to Equation (1):

%Sw_eq_ = (weq − ws)/ws × 100 (1)

where weq represents the weight of the wet biopolymers at final time (24 h) and ws is the weight of the dry biopolymers.

#### 2.2.5. Measurements of Contact Angle

To assess the hydrophilic/hydrophobic nature and wettability of the films, static water contact angle measurements were performed using an optical tensiometer Theta Flow (Biolin Scientific, Gothenburg, Sweden). A 5 μL droplet of double-distilled water was deposited onto the biopolymer surface, and the contact angle was recorded at the initial time (t = 0 s) and every 5 min for a total duration of 25 min. The contact angle analysis was conducted using the OneAttension^®^ software, 3.0. The reported values represent the average of five independent measurements.

### 2.3. Biological Assay

#### 2.3.1. Bacterial Culture and Preparation

*S. aureus* (ATCC 25923) was maintained at a temperature of −80 °C in glycerol stock solution. Bacteria cells were cultured at 37 °C in Luria–Bertani broth (LB). Cultures were grown overnight in LB medium and harvested at the exponential growth phase. The cells were finally resuspended in sterile phosphate-buffered saline (PBS).

#### 2.3.2. Bacterial Adherence Assay

An aliquot of *S. aureus* culture in the exponential phase (1 × 10^6^ CFU·mL^−1^) was incubated in PBS with each biomaterial separately at 37 °C for 24 h with shaking. A control was incubated under the same conditions with no biomaterial. Two washes with phosphate-buffered saline (PBS) were conducted to eliminate any free microorganisms. Finally, to evaluate the microorganisms attached to the biomaterial, the biomaterials were resuspended in clean sterile PBS (500 μL) and vortexed vigorously for 10 min. The number of microorganisms attached to the biomaterial was determined using the microdrop technique, following the methodology outlined by Romeiro [20]. Colony attachment was reported as colony forming units per milliliter (CFU·mL^−1^).

#### 2.3.3. Adhesion Studies by SEM

The adhesion of *S. aureus* to each film was examined using SEM, after fixing the samples with 2.5% glutaraldehyde in phosphate buffer (0.1 M, pH 7.2) for 5 min, followed by two washes with phosphate buffer (0.02 M, pH 7.2). Next a dehydration process was performed, where the samples were incubated in a series of increasing concentrations of cold ethanol for 10 min each. The critical point drying technique was applied. Finally, the samples were sputter-coated with a thin layer of gold for 90 s at a current of 40 mA and a pressure of 0.4 mbar under an argon atmosphere.

#### 2.3.4. Statistical Analysis

All experiments were performed in triplicate in three independent experiments, and the results are reported as the average ± standard deviation. All data were analyzed in GraphPad Prism software^®^, version 8, using a one-way ANOVA test followed by Tukey’s post hoc statistical test. A *p* value < 0.0001 was considered statistically significant.

## 3. Results and Discussion

### 3.1. Formation of Biopolymer Films

**Scheme 2:**
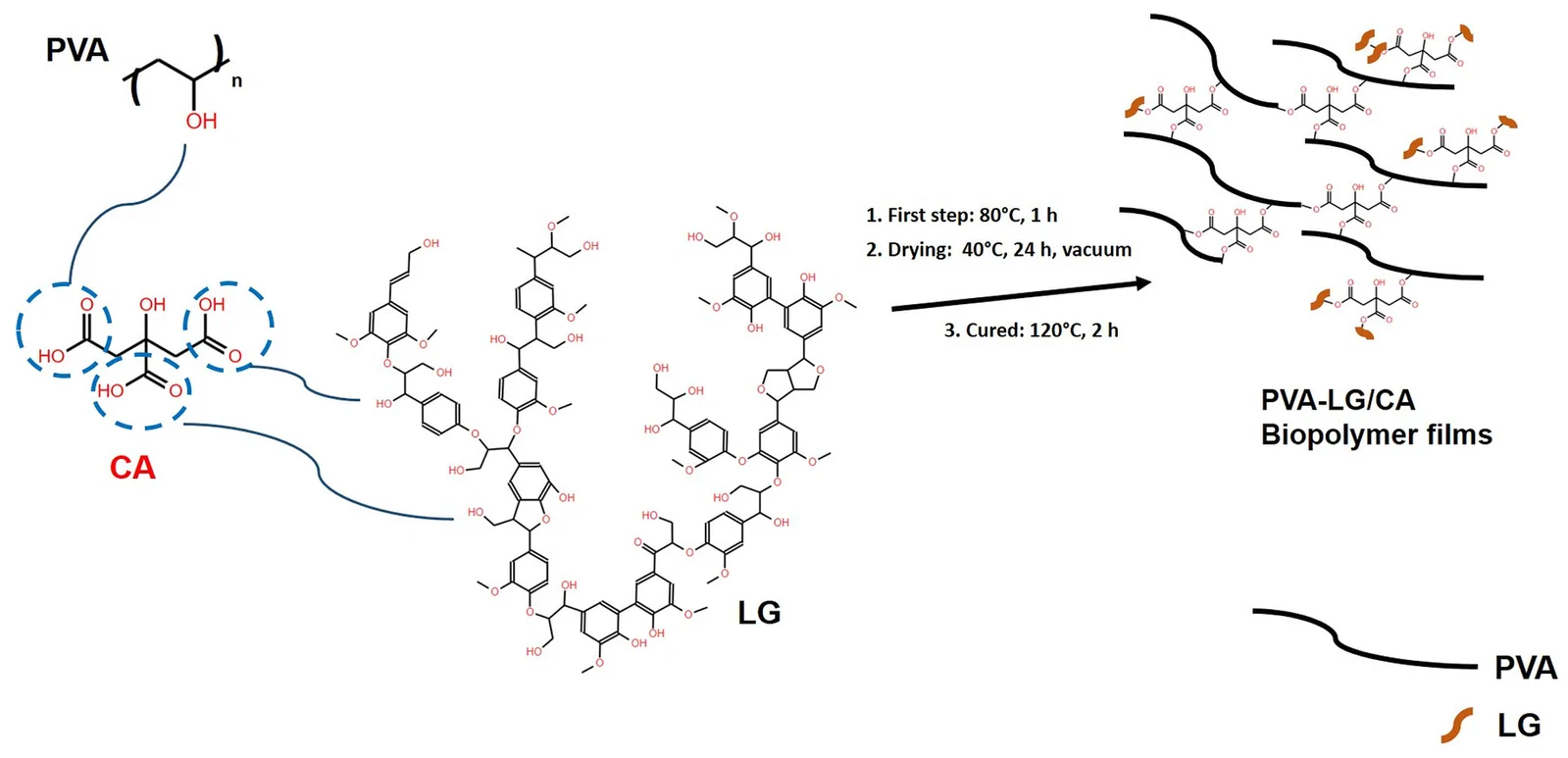
Schematic representation of PVA-LG/CA biopolymer formation via esterification of PVA and LG polymers with CA as a crosslinking agent.

The five kinds of hydrogel materials were successfully synthesized, yielding free standing films. The crosslinking reaction consists of an esterification of citric acid with the hydroxyl (R–OH) groups of PVA and, to a lesser extent, the phenolic groups (Ar-OH) of lignin. The tri-carboxylic structure of CA facilitated the formation of covalent bonds through ester linkages [21]. Because phenolic groups are less nucleophilic than alcoholic groups, the degree of esterification with lignin will be lower [22]. Scheme 2 illustrates the involved reactants, their respective chemical structures, and the proposed reaction mechanism with the final structure of the PVA-LG/CA biopolymer films.

To accurately quantify the LG content in the films, the samples obtained in step C were washed twice with water, and the Folin–Ciocalteu (FC) method was employed to measure the amount of LG released. Table 2 presents the percentage of LG incorporated in the synthesis initially (%LG_initial_ (mass of LG in the synthesis/mass total of PVA, LG and CA)), the percentage of LG released in the washing (%LG_release_ (mass LG released/mass of LG in the synthesis)), and the percentage residual of LG within the final material, post-washing (%LG_residual_ (mass LG remaining in the film/mass total of PVA, LG and CA)). The percentage of LG_residual_ was calculated according to Equation (2):

%LG_residual_ = %LG_initial_ × (1 − (%LG_release_/100)) (2)

Based on the results presented in Table 2, we can observe that the %LG release during the washing step is high, with around 70–90% of the initial LG remaining unbound. Similar behavior was reported for physically crosslinked PVA-LG hydrogels [17].

Additionally, as the concentration of CA increases, the reaction ratio between PVA, LG, and CA increases, decreasing the LG released. However, the B5 biomaterial synthesized with the highest concentrations of LG (10% *w*/*v*) and CA (4% *w*/*v*) shows the lowest residual %LG. This result can be explained by two factors. (1) CA may be more reactive toward PVA than toward LG, likely due to a steric effect. PVA is a linear polymer with a molecular weight (MW: 89,000–98,000 Da) nearly ten times greater than that of LG (MW: 10,000 Da), which has a three-dimensional branched structure. (2) Due to its aromatic characteristics, LG exhibits low solubility in water [23]. Although a homogeneous dispersion is formed in the initial blend, during drying, aggregates and isolated domains may form, which can hinder effective crosslinking, as shown later in the SEM images. When LG agglomerates are formed, the amount available for crosslinking is reduced, resulting in a lower %LG residual. Consequently, within the range of concentrations analyzed, there is a maximum amount of LG that can be incorporated into the film, corresponding in this case to the B4 film, which incorporates 10.2% LG.

### 3.2. Characterization by FTIR-ATR

**Figure 1:**
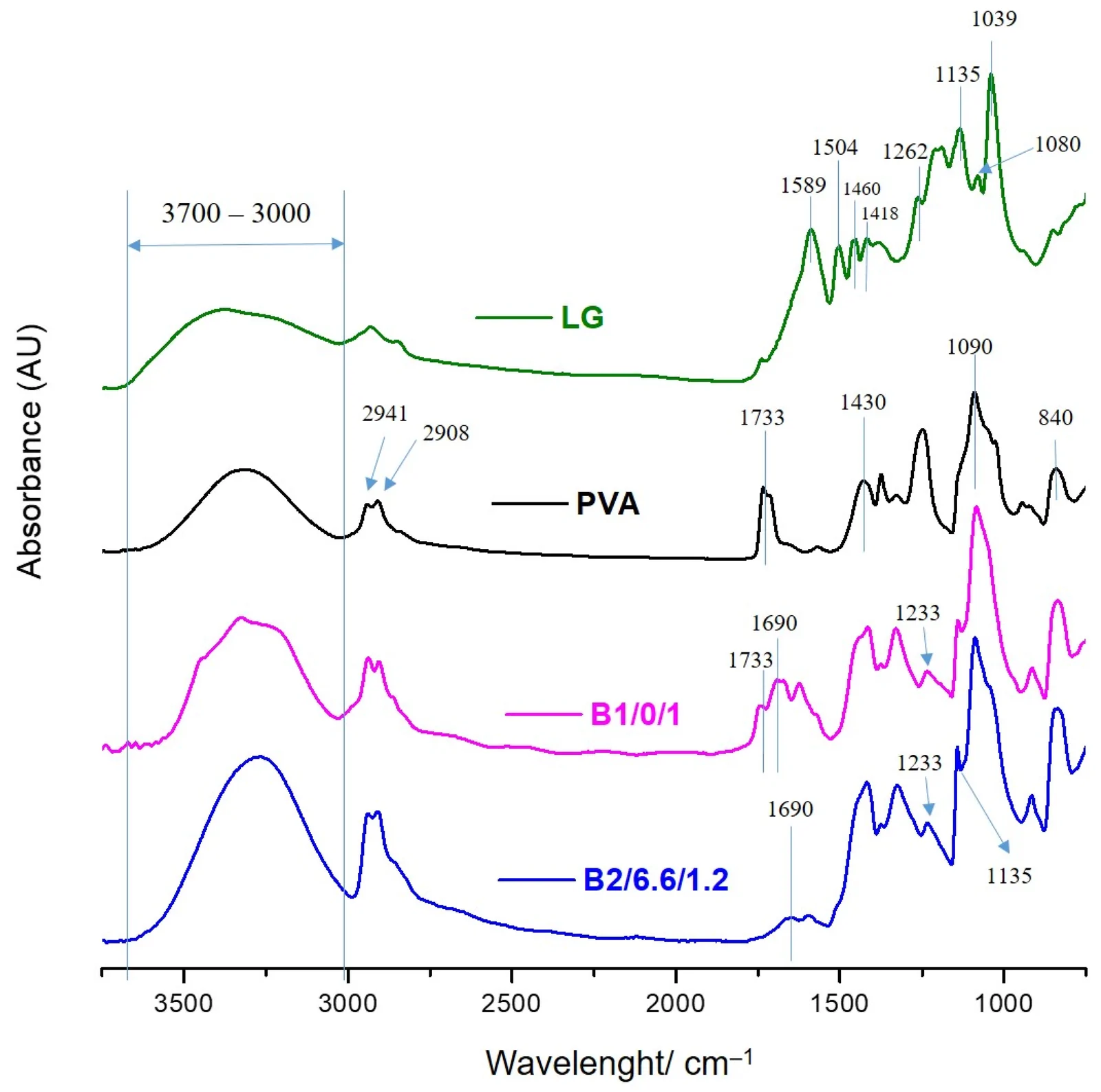
FTIR-ATR spectra of LG, PVA, B1/0/1, and B2/6.6/1.2 biopolymers.

The FTIR-ATR spectra of the LG, PVA, B1/0/1, and B2/6.6/1.2 biopolymers are presented in Figure 1. All spectra exhibit a broad band at 3700–3000 cm^−1^ corresponding to the O-H stretching vibration.

Additionally, the LG spectrum displays two bands at 1589 and 1504 cm^−1^, which are characteristic of the aromatic (Ar) ring C=C vibrations in guaiacyl and syringyl units, respectively; bands at 1460 and 1418 cm^−1^, which correspond to the C–H deformation within the aromatic rings; a band at 1080 cm^−1^, which is associated with C-O stretching in secondary alcohols; an intense band at 1039 cm^−1^ due to C-O stretching in primary alcohols; and a band at at 1262 cm^−1^, which is associated with Ar–O–R stretching in ether and phenol groups. Additionally, a prominent stretching vibration is observed at 1135 cm^−1^, indicative of the presence of ether groups (Ar–O–R) [17,24]. On the other hand, the PVA spectrum exhibits an intense and well-defined band at 1090 cm^−1^, arising from C-O stretching in secondary alcohols; a double band at 2941 cm^−1^ and 2908 cm^−1^, corresponding to aliphatic -CH_2_ stretching; and a band at 840 cm^−1^, correlated with the C-C stretching vibration. Also, a small peak appears at 1733 cm^−1^, originating from the C=O stretching of residual acetyl groups on the PVA molecular chains [25,26]. Similar absorption bands at 3356 cm^−1^ (O–H stretching), 2941 cm^−1^ (C–H from alkyl groups), 1435 cm^−1^ (-CH_2_ bending), and 1094 cm^−1^ (C–O stretching) have been assigned to characteristic functional groups in PVA microfiber mats [27].

Notably, the bands characteristic of ester groups [28] (carbonyl C=O at 1690 cm^−1^ and C-O asymmetric stretching at 1233 cm^−1^) are present in the spectra of both biopolymers (B1 and B2), indicating the presence of ester crosslinks in the films. Moreover, a significant decrease in the intensity of the band at 1039 cm^−1^ (LG primary alcohol) is observed, suggesting that these groups in LG are now participating in ester linkages. The incorporation of LG into the biopolymer is also evidenced by the band at 1139 cm^−1^ corresponding to ether functional groups (Ar-O-C), although this band may also include contributions from C–O–C stretching vibrations associated with ester linkages.

The FTIR-ATR spectra of PVA and LG are provided in the Supplementary Information (Figures S2 and S3), along with a comprehensive analysis that includes the assignment of each band.

### 3.3. Characterization by DSC

**Figure 2:**
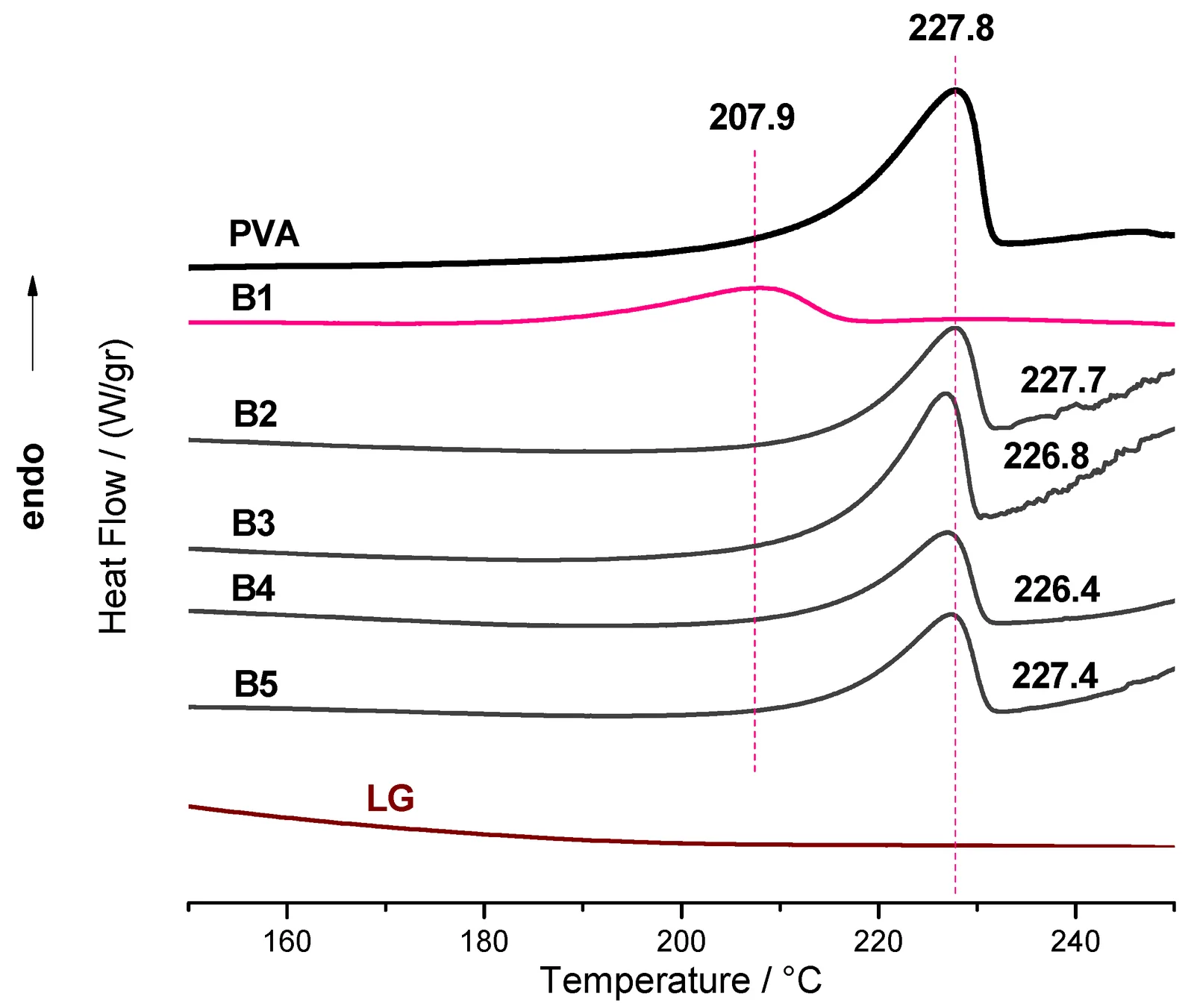
DSC thermograms of pure PVA and LG and the films B1/0/1, B2/6.6/1.2, B3/6.6/1.7, B4/6.6/3.3, and B5/10/4.

The DSC thermograms of PVA, LG, and LG-PVA/CA films are presented in Figure 2. The analysis focuses on identifying changes observed in the melting point (Tm). PVA, a polyol with notable hydrophilicity and water solubility [8], exhibits a semi-crystalline structure. The DSC thermogram of PVA shows an endothermic crystalline peak (Tm) at 228 °C, corresponding to its melting point. By contrast, LG, an amorphous polymer, does not show a crystalline peak but exhibits a characteristic peak at 108 °C, corresponding to its glass transition temperature (Tg), and another at 320 °C, attributed to its initial thermal decomposition [29,30]. (See DSC thermogram, Figure S4).

After analyzing the thermal behavior of the films, it was observed that film B1 exhibits a melting peak (Tm) at 207 °C, which is lower than that of the uncrosslinked polymer, since pure PVA melts at 227.8 °C [31]. This decrease is attributed to a marked loss in polymer crystallinity [32,33], indicating that crosslinking was successful. Lishan Wen et al. [32] reported similar behavior in films based on carboxymethyl chitosan (CMCS)/polyvinyl alcohol (PVA) crosslinked with citric acid. As the %CA increases, the crystallinity of the films decreases compared to the CMCS/PVA blend. This trend is consistent with a reduction in the melting point (Tm). Since CA has inherent crystallinity, when the %CA exceeds 5%, the crystallinity begins to increase.

On the other hand, the films formed by LG and PVA (B2 to B5) exhibit some crystalline domains attributable to partially crosslinked PVA, as evidenced by their DSC thermograms (Figure 2). When a crystalline polymer is combined with an amorphous polymer, and chemical crosslinking occurs between them, the crystallinity of the final material decreases, which is clearly evidenced by a reduction in Tm and melting enthalpy (ΔHm) [17]. In Figure 2, Tm is observed at 227.7 °C, 226.8 °C, 226.4 °C, and 227.4 °C for the B2, B3, B4, and B5 films, respectively. It can be noted that the B2 film, synthesized with the lowest %CA (1.3% *w*/*v*), which resulted in a low degree of crosslinking and minimal residual LG content (residual LG% = 3.3, reported in Table 2), shows a Tm very close to that of pure PVA. By contrast, the B4 film, synthesized with the highest %CA (3.3% *w*/*v*) and the same concentration of LG, incorporates a larger amount of LG (residual LG% = 10.2) due to a higher degree of crosslinking, exhibiting a marked decrease in Tm. According to this data, the PVA-LG/CA films have a non-uniform structure, with distinct domains of PVA and LG that likely formed during the initial synthesis stage due to the poor compatibility between PVA and LG. In the final synthesis step, during washing, a high percentage of non-crosslinked LG (over 70%, see Table 2) is released, resulting in a structure with micro- and macropores. The quantity and size of these pores depend on the concentration of CA used.

### 3.4. Morphology Characterization

**Figure 3:**
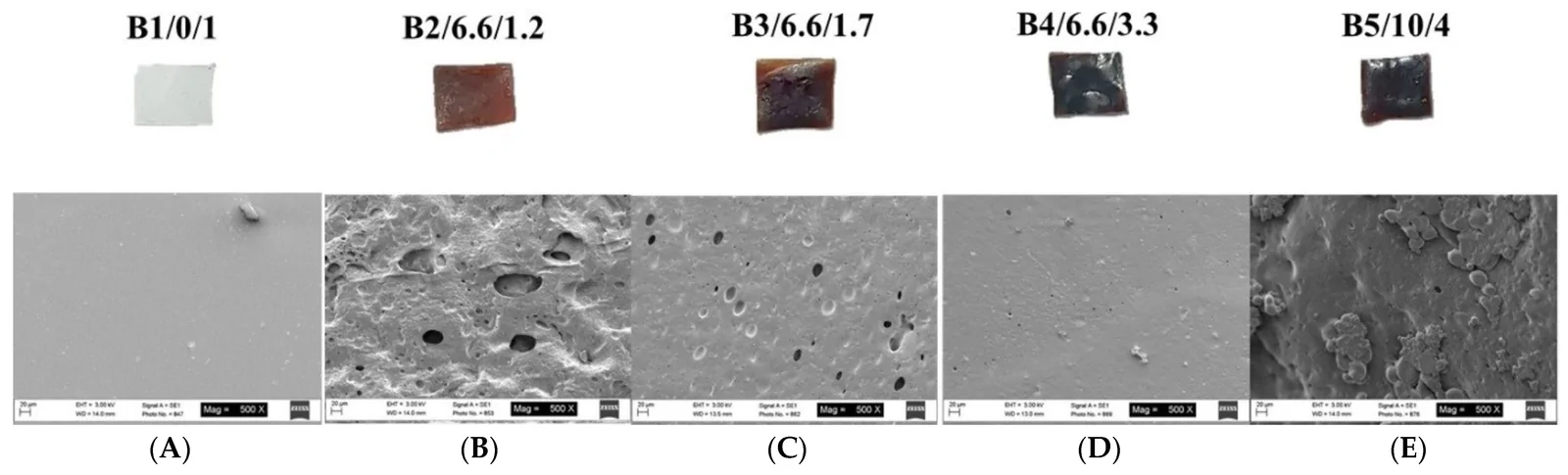
SEM micrographs of the synthesized biomaterials: (**A**) B1/0/1, (**B**) B2/6.6/1.2, (**C**) B3/6.6/1.7, (**D**) B4/6.6/3.3, and (**E**) B5/10/4.

Scanning electron microscopy (SEM) images are shown in Figure 3. The B1/0/1 film possesses the most compact structure, while films incorporating LG exhibit micro- and macroporosity. As revealed by the DSC studies, the significant loss in crystallinity in B1 indicates effective crosslinking between PVA and CA, resulting in a compact, uniform film with minimal surface defects, as shown in Figure 3A. By contrast, films incorporating LG display PVA domains within the structure and limited crosslinking. This reflects the poor miscibility between PVA and LG, leading to small aggregates in the initial blend. After the curing step, the chemically unbound LG is released in the washing step, resulting in a structure with pronounced micro- and macroporosity, as shown in Figure 3B–E.

Biomaterials B2/6.6/1.2, B3/6.6/1.7, and B4/6.6/3.3 were formulated with same concentrations of polyvinyl alcohol (PVA) (10% *w*/*v*) and LG (6.6% *w*/*v*), while varying the citric acid (CA) content from 1.2% to 3.3% *w*/*v*. Increasing the CA concentration resulted in a more uniform film morphology, which can be attributed to the greater number of chemical linkages formed within the PVA-CA-PVA and LG-CA-PVA networks. This increased formation of chemical bonds is also reflected in the higher residual LG content observed from B2 to B4, as shown in Table 2. A greater number of chemical linkages likely reduces the formation of LG-rich aggregates or phase-separated domains in the initial blend, which arise from the poor miscibility between PVA and LG. Consequently, the final films exhibit a more homogeneous morphology, with lower porosity and fewer surface defects. For example, the B2 film, which contains the lowest CA concentration, displays the most heterogeneous and porous structure.

The B5/10/4 film, synthesized with the highest LG (10% *w*/*v*) and CA (4% *w*/*v*) concentrations, exhibits non-uniform LG distribution, with visible surface aggregates. The data reveal a direct correlation between LG concentration and phase instability. LG contents above 6.6% *w*/*v* promote particulate aggregation (Figure 3E), suggesting that the solubility limit in the PVA-CA matrix has been exceeded.

### 3.5. Swelling Behavior

**Figure 4:**
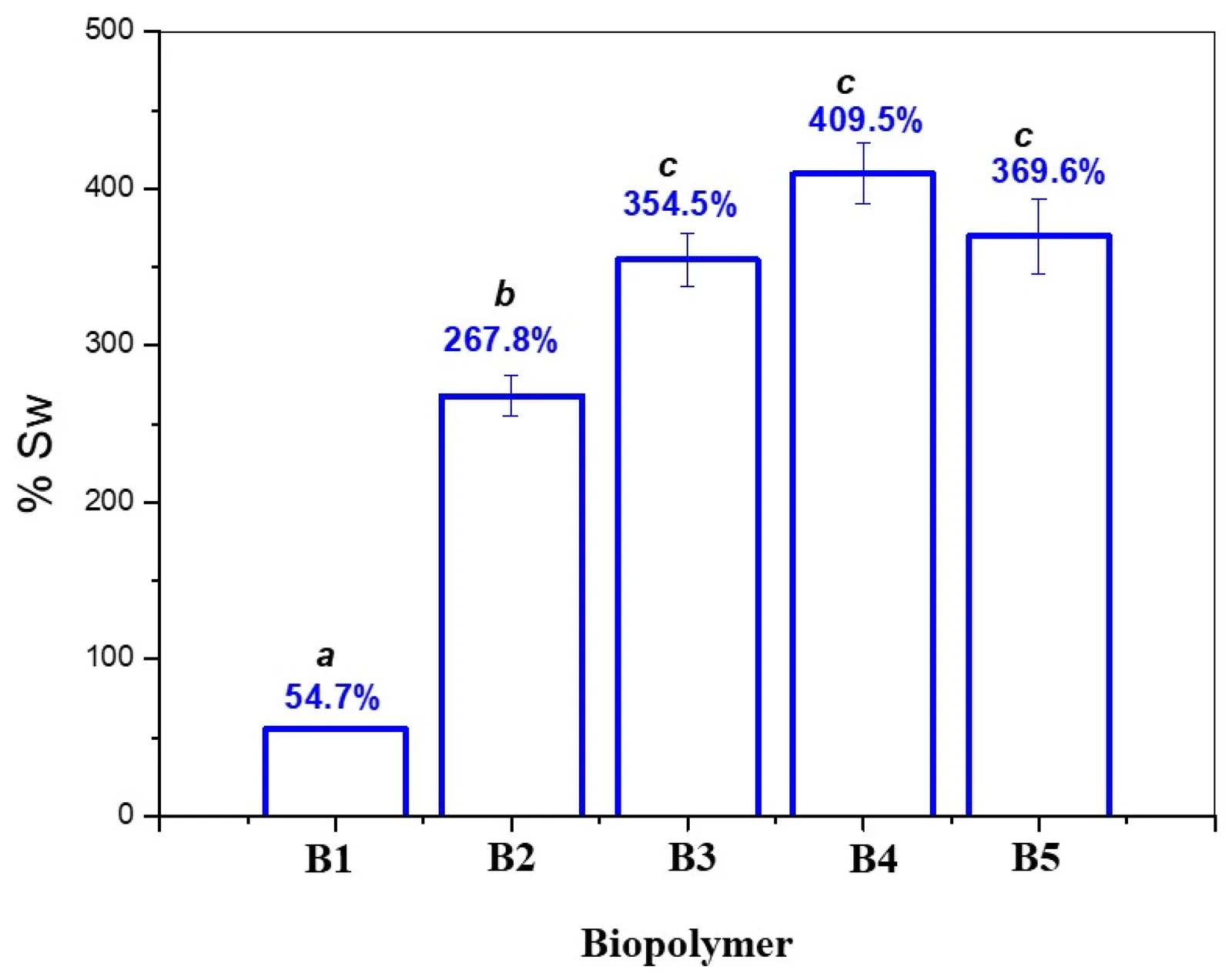
Equilibrium swelling percentages in water for biomaterials B1/0/1, B2/6.6/1.2, B3/6.6/1.7, B4/6.6/3.3, and B5/10/4. All measurements were performed in quadruplicate. Different letters indicate significant statistical differences between materials, ANOVA followed by Tukey’s test (*p* < 0.05).

The swelling capacity of the biomaterials was evaluated by calculating the equilibrium swelling percentage (%Sweq) according to Equation (1). The results are shown in Figure 4.

The ability of these materials to hold water is influenced by both chemical (functional groups and composition) and physical (morphology) factors. B1/0/1 exhibited the lowest swelling capacity (54.7%), which is consistent with its highly compact morphology observed via SEM. By contrast, the incorporation of LG into the films (B2–B5) significantly enhances water uptake, with swelling values 6–8 times higher than those of the LG-free material (B1). This behavior is consistent with the hypothesis proposed above. First, CA shows greater reactivity toward PVA than toward LG, and second, the poor miscibility between PVA and LG promotes the formation of LG-rich domains or aggregates in the initial blend, limiting the formation of chemical linkages between the two polymeric components.

When films B2–B4 are considered, increasing the CA concentration promotes the formation of CA-LG chemical linkages, resulting in a higher percentage of residual LG within the films, as reported in Table 2. Consequently, a greater fraction of LG becomes chemically incorporated into the polymeric structure through CA-mediated linkages, while the amount of LG present as aggregates or phase-separated domains is reduced.

Although the increase in chemical crosslinking with increasing CA content (B2 to B4) would, on purely physical grounds, be expected to produce a more compact network and thus restrict swelling, the opposite trend is observed experimentally: %Sweq increases from 267.8% (B2) to 409.5% (B4) (Figure 4). This apparent discrepancy can be explained by considering that a fraction of the CA molecules remain only partially reacted (not all the three –COOH groups of each CA react), so that free, unreacted carboxylic acid groups persist within the polymeric network. These groups are ionizable and highly hydrophilic, and their relative abundance increases together with the CA content used in each formulation. We therefore propose that the swelling behavior of these films reflects a balance between two opposing effects of increasing CA content: (i) a higher degree of chemical crosslinking, which favors a more compact, less swellable network, and (ii) a higher density of free, hydrophilic carboxylic acid groups, which favors water uptake. For the B2–B4 formulations, the contribution of these hydrophilic groups appears to compensate for, and ultimately outweigh, the swelling restriction imposed by the increased crosslinking density, resulting in the net increase in %Sweq observed across this series.

Consistent with this balance, %Sweq increased significantly from B2 to B3 (%Sw(B3) > %Sw(B2)), whereas no statistically significant differences in %Sweq were observed among B3, B4, and B5 (ANOVA followed by Tukey’s test, *p* < 0.05), suggesting that beyond a certain crosslinking density, the two opposing effects (chemical crosslinking and free carboxylic acid group hydrophilicity) approach a new equilibrium.

Swelling is a critical property of materials designed for controlled drug delivery applications [34]. In this study, the ability of the biomaterial to swell in aqueous media significantly broadens its potential applications. For instance, an antibiotic could be incorporated to achieve a synergistic effect that enhances the desired properties; in this work, these properties include the antifouling and antibacterial performance of the biopolymeric films.

### 3.6. Hydrophilic/Hydrophobic Property

**Figure 5:**
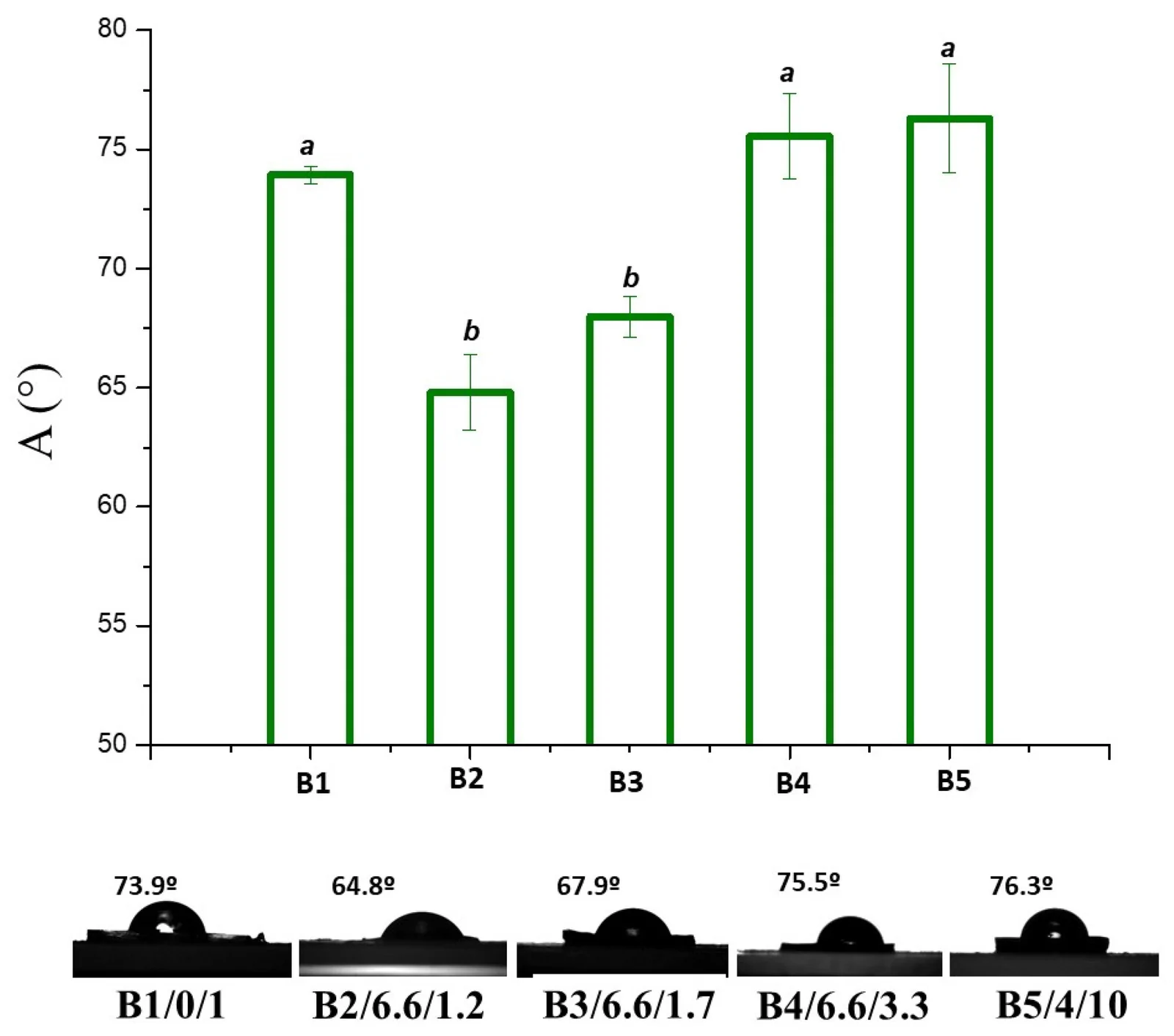
Water contact angles at time = 0 for the studied biomaterials. Different letters indicate significant statistical differences between materials, ANOVA followed by Tukey‘s test (*p* < 0.1).

The contact angle (A) results (Figure 5) show that all materials are hydrophilic (A < 90°). B1, B4, and B5 exhibit the highest values, with no significant differences between them. This slightly more hydrophobic behavior compared to B2 and B3 can be explained by their surface-morphological characteristics, since they all feature a compact structure without visible porosity (see SEM images). By contrast, materials B2 and B3 have a macro- and microporous structure with surface defects.

A wettability study was also conducted, where the water contact angle (A) between the film surface and a water droplet was measured over a 25 min period. Figure S6 presents optical images for the different times analyzed, while Figure S7 shows a graph of A vs. time. It is interesting to note that the films with the lowest contact angles and the most hydrophilic properties display a more pronounced decrease in A (−3.92 °C·min^−1^, −3.19 °C·min^−1^, −2.15 °C·min^−1^, −2.06 °C·min^−1^, and −1.87 °C·min^−1^ for B2, B3, B5, B4, and B1, respectively), indicating a higher wettability rate.

### 3.7. Biological Assay: Anti-Adhesion Assay Using the Microdrop Technique

**Figure 6:**
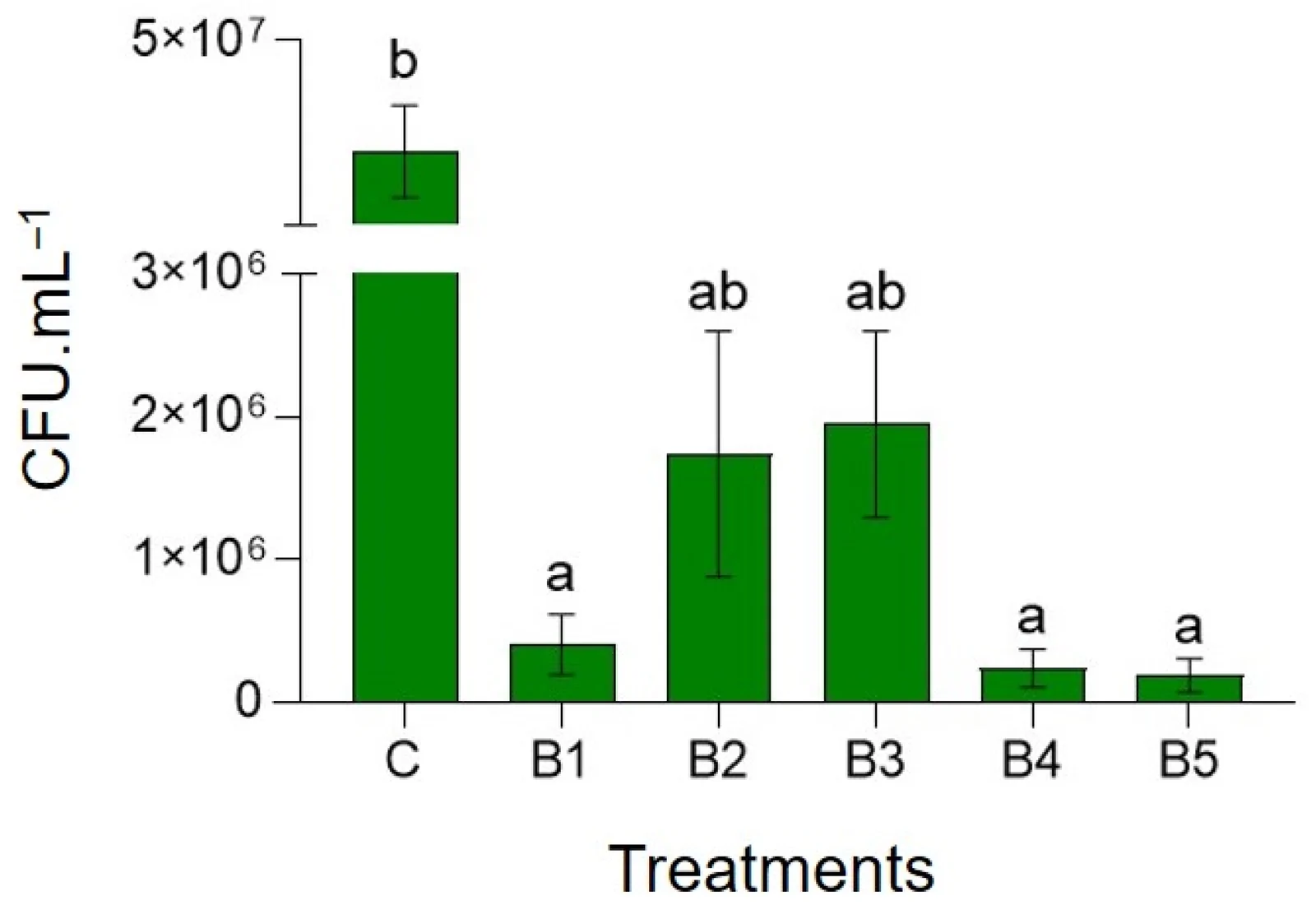
Adhesion of *S. aureus* on each biopolymer. C, control; B1/0/1, B2/6.6/1.2, B3/6.6/1.7, B4/6.6/3.3 and B5/10/4. ANOVA followed by Tukey’s test *p* < 0.0001. Different letters indicate statistically significant differences among treatments (*p* < 0.0001).

The numbers of microorganisms adhered to the surfaces of the films are presented in Figure 6, expressed as colony-forming units per milliliter (CFU·mL^−1^). The treatment without exposure to any material (referred to as the control, C) exhibited a microbial count of 3.5 × 10^7^ CFU·mL^−1^. Also, the PVA-LG formulation without CA is not considered a control because under the synthesis conditions used in the present work, CA is required to obtain a chemically crosslinked PVA-LG film with sufficient stability in aqueous media.

Films B1, B4, and B5 demonstrated similarly low microbial adhesion, with counts of 4.05 × 10^5^ CFU·mL^−1^, 2.39 × 10^5^ CFU·mL^−1^, and 1.89 × 10^5^ CFU·mL^−1^, respectively. This corresponds to a reduction of nearly two orders of magnitude in CFU·mL^−1^ compared to the control. By contrast, films B2 and B3 exhibited higher adhesion of *S. aureus* to their surfaces, although no statistically significant difference was observed between them.

Gram-positive bacteria (*S. aureus*) are more sensitive to citric acid than Gram-negative bacteria (*Escherichia coli*) [27,35]. These results indicate that CA should enhance the anti-adhesive properties of the crosslinked biopolymer films by actively interacting with bacterial cell membranes, disrupting protein and membrane structures and thereby inhibiting bacterial growth. Sabzi et al. [26] reported PVA/CA/Ag NPs nanocomposite hydrogels loaded with ciprofloxacin with effective antibacterial activity against *E. coli* and *S. aureus*. The authors demonstrated the pronounced antibacterial effect of PVA crosslinked with CA (PVA-CA) and PVA loaded with Ag NPs (PVA-Ag). By contrast, pure PVA did not exhibit a detectable inhibition zone for the growth of either microorganism. Wen et al. [32] observed similar behavior in their study. Consequently, CA is a significant factor contributing to reduced bacterial adhesion, although not the only one. Other factors, including hydrophilicity/hydrophobicity, wettability, and morphological characteristics, must also be considered [36].

The wettability of a surface has been extensively reported as a significant factor influencing bacterial adhesion [37]. Zen et al. [36] demonstrated that hydrophobic hydrogels exhibit exceptional antifouling properties, particularly in resisting adhesion by the Gram-negative bacterium *E. coli*. These hydrophobic hydrogels were characterized by high static water contact angles (A > 90°), signifying pronounced surface hydrophobicity—an uncommon feature for conventional hydrogels. This pronounced hydrophobicity was primarily responsible for their superior antifouling capabilities. In our case, B1, B4, and B5 films demonstrate elevated contact angle values, resulting in enhanced hydrophobicity compared to B2 and B3, and consequently exhibiting minimal bacterial adhesion. Additionally, the B2 and B3 films exhibit the highest wettability rates, as shown in Figure S7, demonstrating their greater affinity for water. Moreover, superhydrophobic materials prepared on stainless steel and copper surfaces through various methods have shown good resistance to biofouling [38,39].

**Figure 7:**
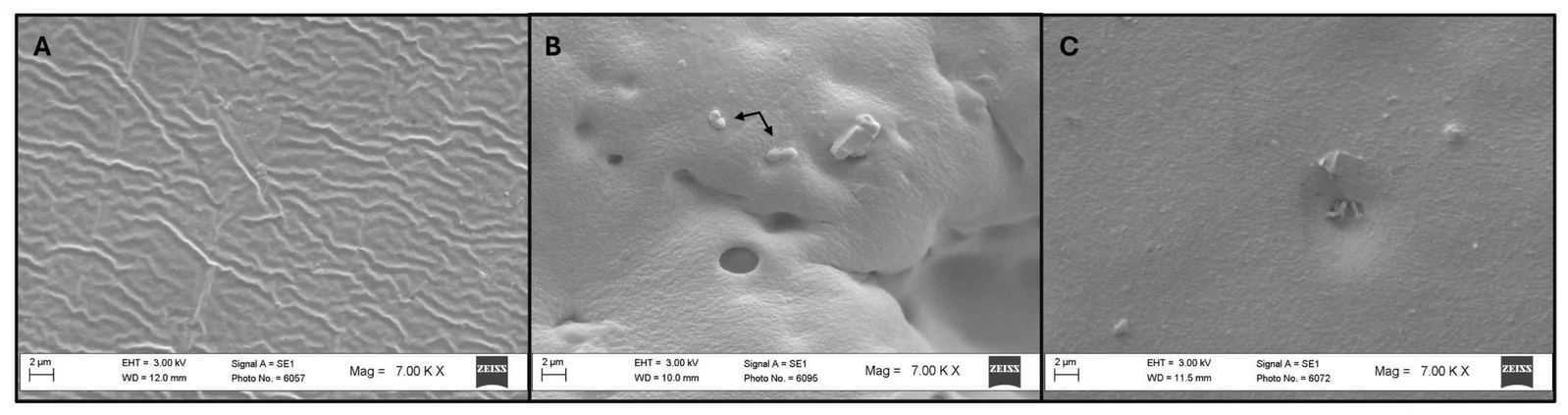
SEM micrographs showing the adherence of *S. aureus* on each biopolymer. (**A**) B1/0/1, (**B**) B2/6.6/1.7, and (**C**) B4/6.6/3.3. The arrows in (**B**) indicate the presence of bacteria on the surface.

Surface morphology is another parameter to consider, which, in our case, is directly related to the hydrophobicity/hydrophilicity of the material. Thus, films with smooth surfaces (B1), minimal surface defects, and very small pores (B4 and B5) exhibit the highest contact angle (A) values and show minimal bacterial adhesion. The opposite is observed for films B2 and B3. This behavior is further confirmed by SEM micrographs, which illustrate the adherence of *S. aureus* on the films (Figure 7). The B1 and B4 films (Figure 7A,C) display no attached microorganisms, correlating with the previously reported colony counts. Conversely, the B3 film (Figure 7B) shows two diplococci of *S. aureus* (indicated by arrows) attached near the pores.

Based on the results presented, it is clear that the composition of LG and CA can be used to the control surface morphology, swelling, and hydrophilic/hydrophobic properties of the biopolymeric films, which directly influences the adhesion of microorganisms. The B4 film was selected as the best candidate for further studies, as it exhibits the highest incorporation of LG, maximum swelling (409.5%), and minimal adhesion of *S. aureus*.

## 4. Conclusions

This study demonstrates the successful development of biopolymeric films composed of PVA-LG crosslinked with CA, tailored to exhibit enhanced anti-adherence properties.

All films exhibited hydrophilic characteristics (A < 90°); however, those with the highest contact angles (B1, B4, and B5) demonstrated superior anti-adhesion properties. Surface hydrophilicity/hydrophobicity is a crucial factor to consider. The incorporation of lignin (LG) significantly improved the water absorption capacity of the films, with swelling values surpassing 350%. This pronounced swelling behavior provides promising opportunities for future research, particularly for the potential loading of water-soluble antimicrobial compounds to enhance or expand the functional properties of materials. Scanning electron microscopy (SEM) analysis provided structural insights, showing that the inclusion of LG induced a porous matrix, while variations in citric acid concentration directly influenced film density and pore architecture. These morphological characteristics were found to significantly impact both the physical properties of the materials and their interaction with microorganisms.

Biological assays demonstrated that the anti-adhesion properties of the biopolymeric films against *S. aureus* were directly influenced by their composition. Films with lower concentrations of LG and CA showed increased bacterial adhesion due to their porous structure, while those with higher concentrations exhibited reduced bacterial attachment. This suggests that the films can be engineered to achieve specific antimicrobial outcomes, either preventing biofilm formation or supporting controlled bacterial growth, depending on the application.

The present study focused on the Gram-positive pathogen *S. aureus*. Extending this evaluation to Gram-negative bacteria such as *E. coli*, which possess a distinct outer membrane and are generally less susceptible to citric acid-based antibacterial mechanisms [27,35], is an important direction for future work.

Building on these results, future work will focus on depositing the optimized PVA-LG/CA formulation (B4) as a coating on clinically relevant substrates (e.g., catheter tubing) and evaluating coating adhesion, stability, and antibacterial performance under conditions that more closely resemble real device applications.

In parallel, further optimization of the material itself will be explored, including the incorporation of compounds of interest such as essential oils [40] or nanoparticles [41] and evaluating their antibacterial properties.

## Figures and Tables

**Table 1:** Composition of PVA, LG, and CA in each biopolymeric film.

Film ^1^	PVA (% *w*/*v*)	LG (% *w*/*v*)	CA (% *w*/*v*)
B1/0/1	10	-	1
B2/6.6/1.2	10	6.6	1.2
B3/6.6/1.7	10	6.6	1.7
B4/6.6/3.3	10	6.6	3.3
B5/10/4	10	10	4

^1^ The films are named: B (number of biopolymer)/LG%CA%, since the PVA% is maintained at 10%.

**Table 2:** Lignin percentage (%) initial, release in the first washing ^1^, and residual for each film.

Film	% LG_initial_	% LG_release_	% LG_residual_
B1/0/1	0	0	0
B2/6.6/1.2	37.1	91.1	3.3
B3/6.6/1.7	36	79.4	7.4
B4/6.6/3.3	33.1	69.2	10.2
B5/10/4	41.7	88.4	4.8

^1^ No LG was detected in the second wash using the FC method.

## Data Availability

The data supporting the findings of this study are available within the article and Supplementary Materials. Additional raw data are available from the corresponding author upon reasonable request.

## Supplementary Materials

The following supporting information can be downloaded at https://www.mdpi.com/article/10.3390/polym18182244/s1, Equations for calculating %LG residual; Figure S1. Calibration curve of LG using RC regent; Figure S2. FTIR-ATR spectrums for PVA; Figure S3. FTIR-ATR spectrums for LG; Figure S4. DSC thermogram of LG; Figure S5. Swelling of the biopolymers in: Ethanol, Dimethylformamide, Toluene; Figure S6. (A) Recorded of photographs of water contact angle from initial (time = 0 s) each 5 min over a 25-min period for B1/0/1, B2/6.6/1.2, B3/6.6/1.7, B4/6.6/3.3 and B5/10/4 biomaterial. (B) A vs. time graph; Figure S7. A vs. time graph. Measurements are reliable up to 15 min, as longer times result in material deformation and water droplet evaporation.
