# Supplementary material for: Polyvinyl Alcohol/Lignin Composite Films Crosslinked with Citric Acid: Physicochemical Properties and Anti-Adhesive Activity Against Staphylococcus aureus
Source: Polymers (Basel). 2026 Sep 15;18(18):2244. doi: 10.3390/polym18182244 (PMC13611098; doi:10.3390/polym18182244)
Supplement: Supplementary file 1 [file polymers-18-02244-s001.zip › polymers-4545113-supplementary.pdf]

SUPPLEMENTARY INFORMATION

Equations to calculation %LG residual

$$\%\text{LGresidual} = \%\text{LG initial} \times (1 - \frac{\%\text{LGrelease}}{100})$$

$$\%\text{LGresidual} = \%\text{LG initial} \times (1 - \frac{\text{mass LG released}}{\text{mass LG SYNTHESIS}})$$

$$\%\text{LGinitial} = \frac{\text{mass LG synthesis}}{\text{mass total of PVA, LG and CA}} * 100$$

$$\%\text{LG release} = \frac{\text{mass LG released}}{\text{mass LG SYNTHESIS}} * 100$$

$$1 = \frac{\text{mass LG released}}{\text{mass LG SYNTHESIS}} + \frac{\text{mass LG re}\mathit{SIDUAL}}{\text{mass LG SYNTHESIS}}$$

$$\%\text{LGresidual} = \frac{\text{mass LG synthesis}}{\text{mass total of PVA, LG and CA}} * 100 * (1 - \frac{\text{mass LG released}}{\text{mass LG SYNTHESIS}})$$

$$\%\text{LGresidual} = \frac{\text{mass LG synthesis}}{\text{mass total of PVA, LG and CA}} 100 * (\frac{\text{mass LG re}\mathit{SIDUAL}}{\text{mass LG SYNTHESIS}})$$

$$\%\text{LGresidual} = (\frac{\text{mass LG RESIDUAL}}{\text{mass total of PVA, LG and CA}}) * 100$$

Figures

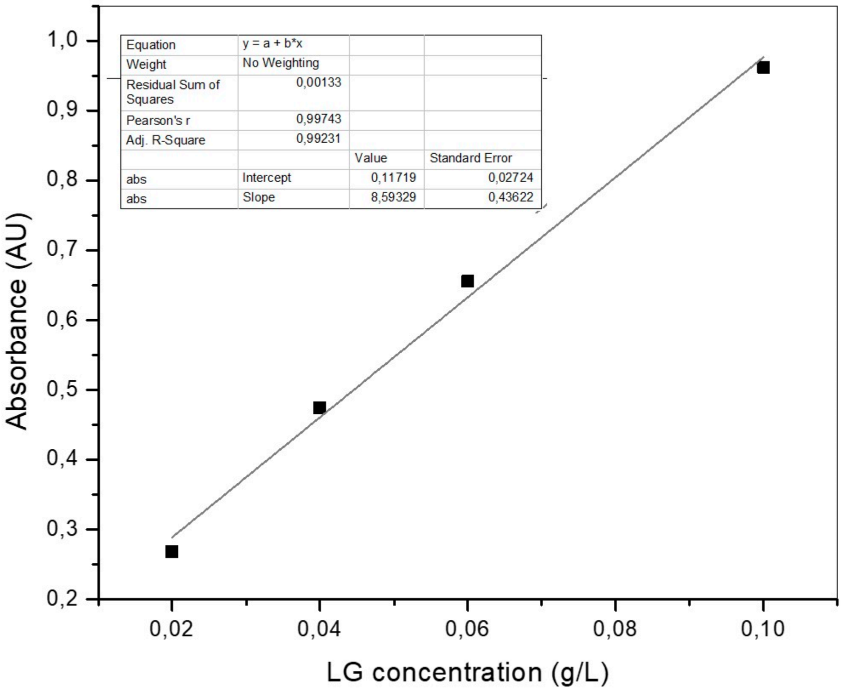

Figure S1. Calibration curve of LG using RC regent

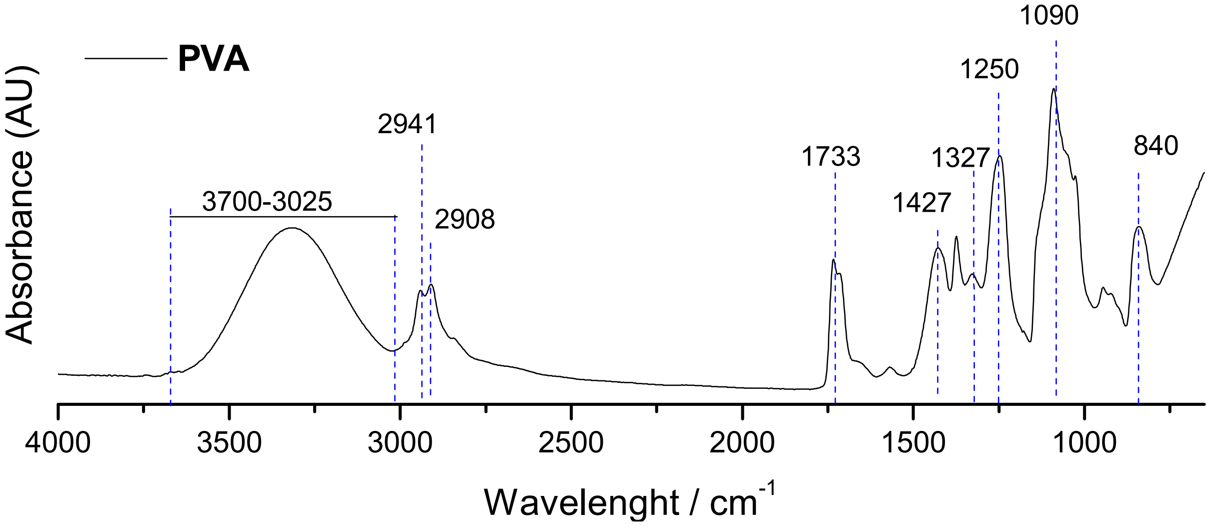

Figure S2. FTIR-ATR spectrums for PVA.

Band Assignment

3700-3025 cm^{-1} / -OH stretch vibration

2941 and 2908 cm^{-1} / alkyl asymmetric C-H stretching vibration

2845 cm^{-1} / C-H symmetric stretch vibration

1733 cm^{-1} / C=O residual stretch vibration

1427 cm^{-1} / -CH_2 bending vibration

1327 cm^{-1} / -C-OH bending in of plane (secondary alcohol)

1250 cm^{-1} / C-O stretching asymmetric stretching (ester units)

1090 cm^{-1} / C-H y C-O stretching vibration (secondary alcohol)

840 cm^{-1} / C-C and C-O stretching vibration

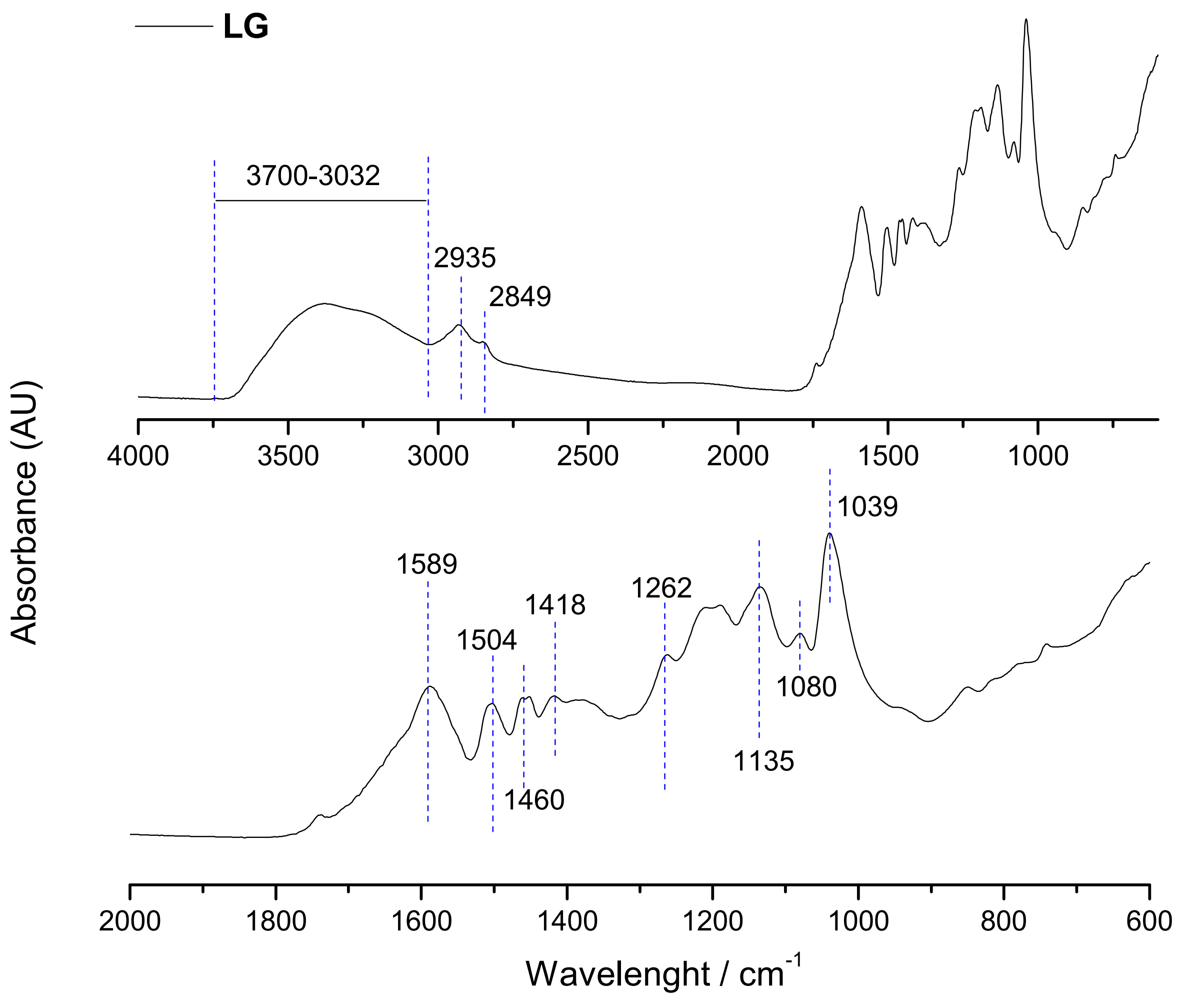

Figure S3. FTIR-ATR spectrums for LG

Band Assignment

3700-3032 cm^{-1} / -OH stretch vibration

2935 cm^{-1} / C-H aromatic stretching vibration

2849 cm^{-1} / C-H symmetric stretch vibration

1589 and 1504 cm^{-1} / C=C aromatic ring stretching (guaiacyl/syringyl units)

1460 and 1418 cm^{-1} / C–H deformation in aromatic rings

1262 cm^{-1} / Ar–O–R stretching (ethers and phenols)

1135 cm^{-1} / Ar–O–R ether stretching vibration

1080 cm^{-1} / C-O stretching secondary alcohol

1039 cm^{-1} / C-O stretching primary alcohol

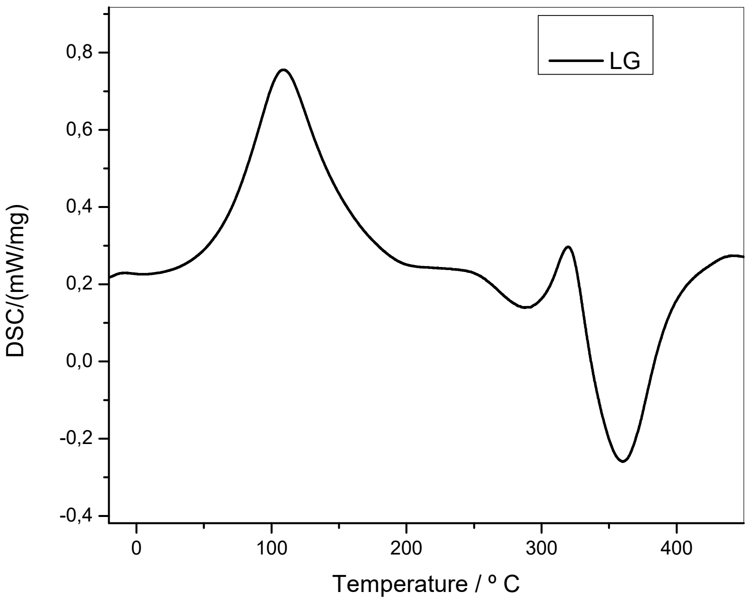

Figure S4. DSC thermogram of LG

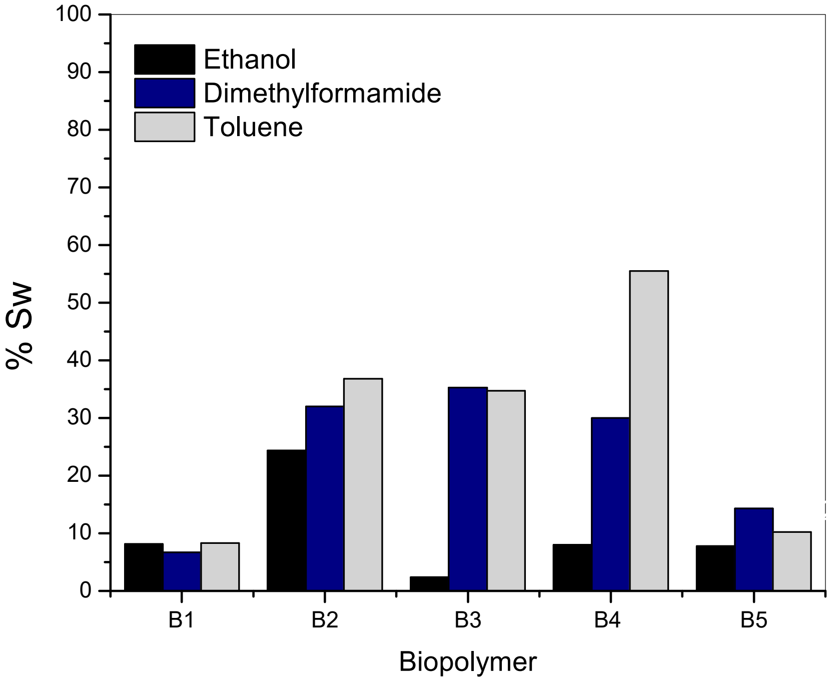

Figure S5. Swelling of the biopolymers in: Ethanol, Dimethylformamide, Toluene.

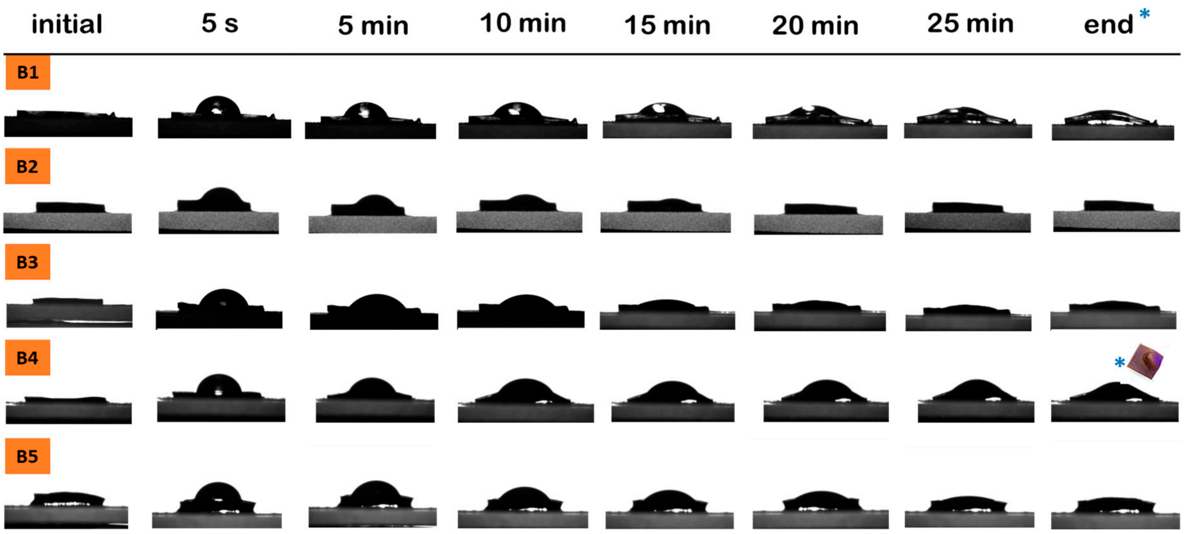

**after a 25-minute period, the material was superficially dried, and subsequent photographs were taken. **An additional optical image*

Figure S6. A) Recorded of photographs of water contact angle from initial (time=0 s), each 5 minutes over a 25-minutes period for B1/0/1, B2/6.6/1.2, B3/6.6/1.7, B4/6.6/3.3 and B5/10/4 biomaterial. B) A vs. time graph.

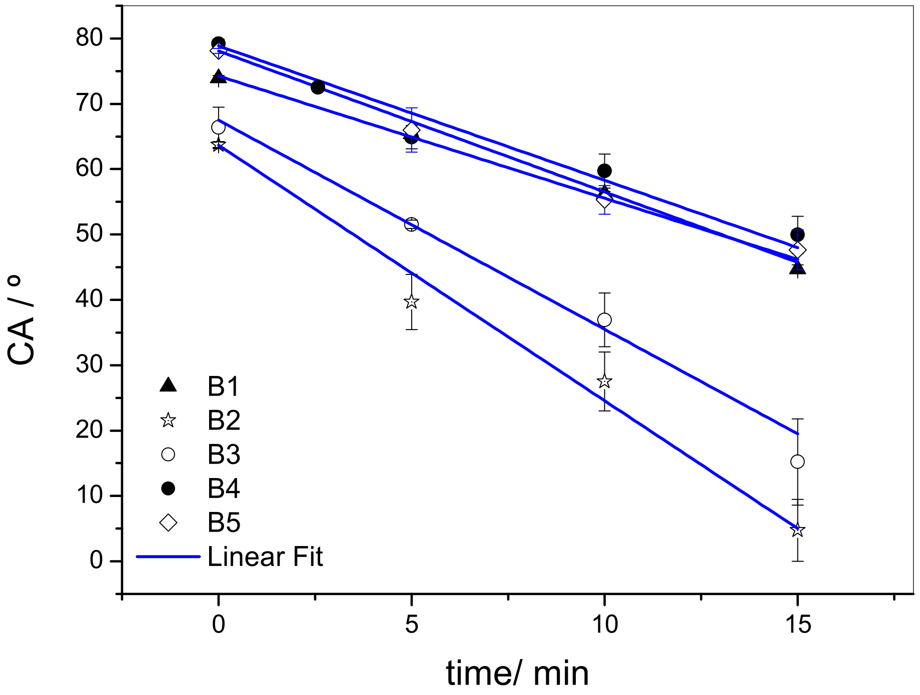

Figure S7. A vs. time graph. Measurements are reliable up to 15 minutes, as longer times result in material deformation and water droplet evaporation.
